# Wanted—A Hospital Administrator

**Published:** 1909-06-12

**Authors:** 


### HOSPITAL ADMINISTRATION.

### CONSTRUCTION AND ECONOMICS.

### WANTED—A HOSPITAL ADMINISTRATOR.

#### THE JERSEY HOSPITAL CASE.

The Jersey Hospital libel case, concluded in Jersey last month after a hearing which lasted many days, possesses features of more than local interest. The plaintiffs were the Committee of Public Assistance appointed by the States of Jersey, and are the managers of the Jersey General Hospital; the defendant, Mr. H. G. Mirehouse, is a resident in Jersey, and had published in the local press towards the end of last year a letter containing grave reflections upon the management and administration of the hospital. In view of the result of the case, it is proper that the parts of the letter alleged by the plaintiffs to be defamatory should be set out in full:

(*a*) Would you be surprised to hear, sir, that instances have been known of patients losing a member by—no, not the neglect—but the insufficient technical and sanitary knowledge of attendants?

(*b*) Is it a fact that poor patients, perhaps during their last moments upon earth, may be troubled by the senseless and merely curious visits of casual acquaintances or stray visitors?

(*c*) Would you be surprised to hear that patients suffering from phthisis—that most insidious and highly contagious or infectious scourge of modern as of ancient times—are isolated by being placed in a separate ward, just across the passage, and that tiny children as well as patients wander hither and thither *à volonté*? This, at least, is not the fault of the medical or nursing staff, but the grievous want of proper accommodation.

(*d*) I have heard tell of a poor sufferer dying of consumption who protested that he could not eat beef and vegetables, but that, none the less, no alteration was made in his diet.

(*e*) I have heard tell of a patient who, after an operation, was attended by a nurse who had charge of a diphtheria case, with the natural result.

(*f*) Also that contagion has been, on other occasions, conveyed by insufficient or sufficient (*sic*) segregation or disinfection.

In view of the importance of the matters in issue the Attorney-General was, on the application of the plaintiffs, made a party to the case as representing the public, a form of procedure with which we are unfamiliar in this country, but which afforded the Attorney-General an opportunity of summing up the case impartially to the Court in his concluding speech—a function which would, of course, in England be performed by the presiding judge in his address to the jury. In Jersey, however, the case was heard before the Bailiff and two Jurats only.

Few graver libels than those indicated above could possibly be published on the managers of a hospital at the present day. It is sufficient to quote the innuendo which the plaintiffs themselves sought to place upon the words in their original "remonstrance" addressed to the Court. They complained that the words were "calculated to shake the confidence reposed in the said committee in leading to believe that they have neglected to discharge the duties vested in them by the States of this Island in assuring the proper administration and government of the said public institution, and that they have allowed a state of things to exist which jeopardises the health and places in peril the lives of inmates entrusted to the care of the staff of the said hospital." Yet the judgment of the Court found that the libels (*a*), (*b*), (*e*), and (*f*) had been proved in substance and in fact; that (*c*) had been proved in substance and in fact, except in regard to the tiny children, and that the defendant had alone failed to prove (*d*). And with regard even to (*d*), it is clear, from his concluding speech, that the Attorney-General regarded strict legal proof of the allegations as alone wanting. Having regard to these findings, the final result of the proceedings, so far as the defendant is concerned, is surprising. He is fined the nominal sum of one shilling, and has to pay the Attorney-General's costs; he is condemned to pay £1 damages to the plaintiffs, and each party is left to bear their own costs. The pitfalls of a plea of justification in a libel case are well known, and the position of a defendant who fails to prove all his charges up to the hilt is never an enviable one, but surely a procedure which produces the results in Mr. Mirehouse's case is open to serious criticism. That no part of his costs should be borne by the persons whom he has charged with, and proved to be guilty of, negligence and incompetence in discharge of their public duties, is hard enough, but it is deplorable that he should be made to bear the costs of the representative of the public, to whom he has by his action rendered such great and signal service.

The state of affairs disclosed by the evidence given in the case is such as to demand instant and ruthless action by the people of Jersey. It must be remembered that the hospital is a public institution, subsidised with public money, and, it is to be assumed, more open to direct public control than the majority of large hospitals in this country. The greater the trust, the greater the responsibility, and we do not doubt that after the lamentable disclosures of the recent trial, a strong effort will be made, not only to rid the hospital of its existing management, but also to bring it in every respect into line, as regards efficiency, scientific administration, and public utility, with the best institutions of the same kind in the British Isles. If this desirable consummation is brought about the sacrifices which Mr. Mirehouse has been forced to make in defence of his public-spirited and courageous action will not have been in vain, and a blot will have been removed from the fair fame of a progressive community which its public men should never have allowed to rest upon it.

